# Characterization of Activated Carbon from Rice Husk for Enhanced Energy Storage Devices

**DOI:** 10.3390/molecules28155818

**Published:** 2023-08-02

**Authors:** Meir S. Yerdauletov, Kuanysh Nazarov, Bagdaulet Mukhametuly, Mukhtar A. Yeleuov, Chingis Daulbayev, Roza Abdulkarimova, Almas Yskakov, Filipp Napolskiy, Victor Krivchenko

**Affiliations:** 1 Institute of Nuclear Physics, Almaty 050032, Kazakhstan; 2 Joint Institute for Nuclear Research, 141980 Dubna, Russia; 3 Faculty of Physics and Technics, L.N. Gumilev Eurasian National University, Astana 010008, Kazakhstan; 4 Faculty of Physics and Technology, Al-Farabi Kazakh National University, Almaty 050040, Kazakhstan; 5 Bes Saiman Group, Almaty 050057, Kazakhstan; 6 National Laboratory Astana, Nazarbayev University, Nur-Sultan 010000, Kazakhstan; 7 Battery Prototyping Laboratory, Dubna State University, 141982 Dubna, Russia; victi81@mail.ru

**Keywords:** activated carbon, chemical activation, lignocellulosic biomass, X-ray diffraction

## Abstract

The production of activated carbon (AC) from lignocellulosic biomass through chemical activation is gaining global attention due to its scalability, economic viability, and environmental advantages. Chemical activation offers several benefits, including energy efficiency, reduced carbonization time, and lower temperature requirements. In this study, potassium hydroxide (KOH) was employed for chemical activation, resulting in activated carbon with a high specific surface area of ~3050 m^2^/g. The structural analysis revealed the presence of graphitized carbon in the activated carbon matrix, accounting for over 15%. The X-ray diffraction (XRD) technique was employed to investigate the activated carbon derived from rice husk (RH). The potential applications of activated carbon obtained from rice husks through chemical activation were explored, including its use for heavy metal removal, elimination of organic pollutants, and as an active material in hybrid energy storage devices. Furthermore, a scaling methodology for the production of activated carbon was proposed, facilitating its industrial implementation.

## 1. Introduction

Activated carbon (AC) is widely utilized in various applications, such as water purification, air filtration, and as an active material for energy storage devices (batteries and supercapacitors) [1]. ACs are carbon-based materials that have a highly porous structure and, therefore, a high specific surface area, which affects surface interactions and diffusion within their structures [2]. The porous structure, particularly the size and distribution of pores, plays a vital role in determining the functionality and applications of ACs. These properties are influenced by the choice of preparation method and precursors, and understanding the interconnected factors that determine these properties is crucial for tailoring ACs to meet specific application requirements. While ACs can be synthesized from various precursors, including agricultural waste, nevertheless, coal is commonly used as the precursor for commercial ACs.

In recent years, there has been a growing interest in developing and adopting technologies for obtaining Acs from lignocellulosic renewable biomass or agricultural waste, such as rice husks, coconut and walnut shells, barley straw, and others [3,4,5,6]. This interest is driven by several factors, including the increasing volume of industrial waste, the need for sustainable development, and the quest for alternative energy sources. Converting agricultural waste into activated carbon offers an appealing solution, as it addresses waste disposal issues while yielding a valuable product with diverse applications. The use of agricultural waste as a precursor material for ACs offers several advantages over traditional carbon precursors. Firstly, it reduces dependence on petroleum products, since agricultural waste is a renewable resource. Additionally, agricultural waste is often unrefined, non-toxic, and free from chemical additives, minimizing the environmental impact. However, the process of obtaining activated carbon from agricultural waste also poses challenges and limitations. The diverse composition of agricultural waste can influence the quality and properties of the resulting activated carbon. Furthermore, optimizing the activation process for different types of agricultural waste requires further research and development.

While several studies have investigated the impact of processing conditions on the properties of activated carbon derived from specific biomass sources and have developed predictive equations for AC properties, there remains room for improvement in the field of biowaste-derived activated carbon synthesis. The utilization of X-ray scattering methods allows for the study of the nanoscale pore sizes present in ACs, providing valuable insights into their porous structure [7,8]. By comparing the experimental data obtained from XRD with results from X-ray photoelectron spectroscopy (XPS) and nitrogen adsorption/desorption measurements, we can comprehensively analyze the porous structure of ACs derived from rice husks. It is worth noting that while our previous studies have demonstrated the potential of ACs derived from rice husks and walnut shells as active materials for lithium-ion batteries and supercapacitors [9,10,11,12,13], a detailed examination of the porous structure is essential, considering the influence of the production process and precursor choice.

The objective of this study is to investigate and characterize the porous structure of ACs derived from rice husks using XRD technique, and to compare the obtained results with XPS and nitrogen adsorption/desorption measurements. By gaining a deeper understanding of the porous structure and properties of biowaste-derived activated carbon, we aim to contribute to the development of more efficient and sustainable AC materials for various applications.

Due to the large surface area and the presence of electronic conductivity, ACs have found application in supercapacitors. The desire to increase the specific energy while maintaining a high discharge power density has led to the creation of hybrid energy storage, such as lithium-ion capacitors (LICs), in which the positive electrode is formed from AC [14,15]. At the same time, the increase in the specific characteristics of LICs is directly related to the development and implementation of ACs with a large specific surface area.

Therefore, in this work, electrochemical characteristics of synthesized activated carbon in lithium-ion liquid electrolytes were studied. Tests were carried out in symmetrical coin cells, as well as in coin cells with a lithium anode. The specific capacitance of the synthesized material was shown to be 140 F/g. At the same time, the results of cycling against a lithium anode showed that the activated carbon-based electrodes have high capacitive characteristics even after 10,000 cycles. We believe that proposed material is promising for use in hybrid energy storage systems such as lithium-ion or lithium-metal capacitors.

## 2. Results and Discussion

### 2.1. Analysis of Material Morphologies, Structures and Chemical States

#### 2.1.1. SEM

**Figure 1:**
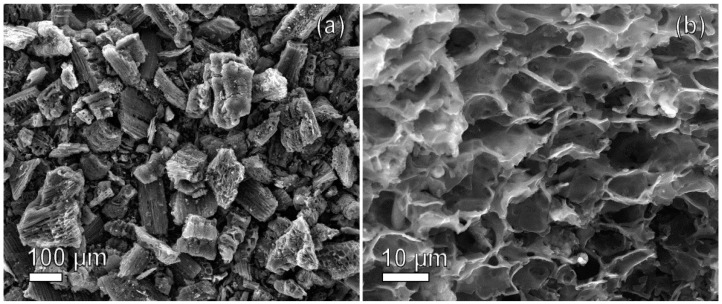
Scanning electron microscope microphotographs of RH-derived activated carbon, captured at (**a**) 100× magnification and (**b**) 1400× magnification (more detailed image is in Supplementary Materials).

Figure 1 shows scanning electron microscope (SEM) micrographs of carbon materials after the thermochemical activation of rice husks. The images (Figure 1a,b) reveal a well-developed porous structure in the carbon material obtained through the process of carbonization–graphitization at 750 °C, followed by thermochemical activation at 850 °C using KOH. The sample exhibits numerous macropores distributed uniformly across the entire surface. However, micro- and mesopores are not discernible at the current magnification level on the SEM images. The porous structure of the carbonized–graphitized and activated rice husk powder was further analyzed using the Brunauer–Emmett–Teller method. The maximum specific surface area achieved through carbonization–graphitization at 750 °C was 210 m^2^/g. However, through subsequent thermochemical activation, the surface area exhibited a significant enhancement, reaching 3050 m^2^/g. This signifies an extraordinary increase by over 14 times in the surface area, highlighting the effectiveness of thermochemical activation in enhancing porosity.

#### 2.1.2. Raman Spectral Mapping of the Activated Rice Husk

**Figure 2:**
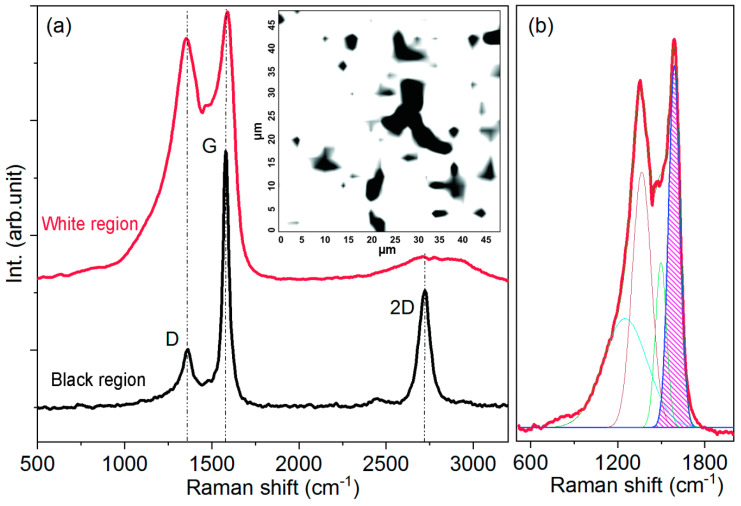
(**a**) Raman spectrum of the graphitized area of the sample (white region on spectral map), Raman spectrum of amorphous carbon (black region on spectral map); inset in (**a**) Raman spectral map of the activated rice husk collected within 50 × 50 µm sample area; (**b**) deconvolution of Raman spectrum of activated carbon.

The structural features of the activated rice husk can be verified through Raman spectra (Figure 2). The Raman spectra clearly display three prominent modes: mode D at 1361 cm^−1^, mode G at 1580 cm^−1^, and mode 2D at 2717 cm^−1^. These modes are widely recognized as characteristic features of graphite [16]. The presence of the D mode is usually associated with the lattice defects of different nature, and the 2D mode corresponds to the second order of the D mode [17,18]. The relative intensity ratio between peak G (IG) and peak 2D (I2D) may provide an estimation of structural imperfection of graphite crystals [19]. According to research conducted in [20], the D/G peak intensity ratio (ID/IG) is known to be an indicator of graphitization, where a smaller ID/IG ratio indicates a higher degree of graphitization [21,22].

In order to investigate the variation in the activated carbon structure, Raman mapping was carried out. The mapping area was 50 × 50 µm. The laser spot diameter was 2 µm, and the mapping step was also 2 µm. Thus, the selected area comprised 25 × 25 points in which the Raman spectra were collected. The results of Raman mapping are presented in Figure 2a (inset) as a two-color scheme. The Raman spectra corresponding to black and white regions are shown in Figure 2. It can be seen that the black regions indicate the locations of graphitic structure, while the white regions indicate the presence of amorphous carbon. According to the Raman mapping, the graphitic structure constitutes approximately 15% of the total volume in the activated carbon matrix.

The deconvolution of the Raman spectrum from white region also revealed two modes near 1200 and 1500 cm^−1^ (Figure 2b). It may be assumed that these modes are related to variation in ratio between sp, sp2 and sp3-hybridized carbon atoms and/or clustering [23] after the process of thermochemical activation. For instance, the presence of polyenes in the annealed biocarbon was also observed in [24]. However, this assumption should be investigated in more detail, which is beyond the scope of this work.

#### 2.1.3. XPS

**Figure 3:**
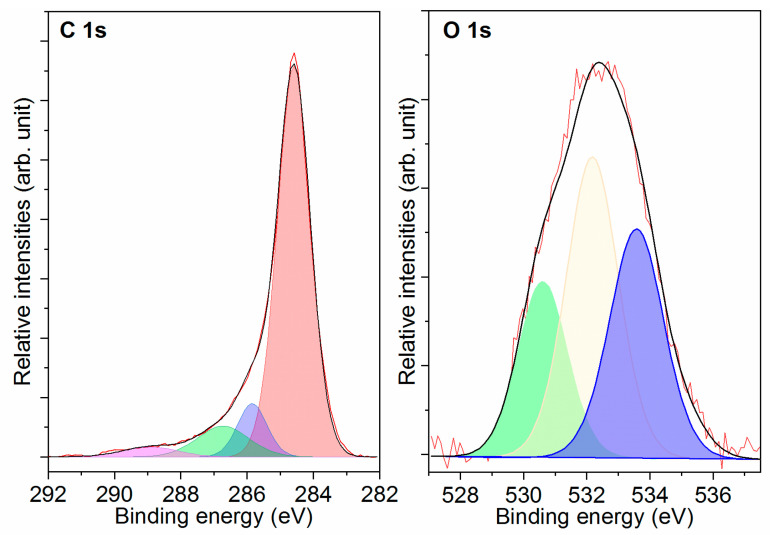
Component peak-fitting of XPS spectra for activated carbon derived from rice husk.

The X-ray photoelectron spectroscopy (XPS) high-resolution data for the obtained samples are presented in Figure 3. The presence of characteristic peaks in the sample indicates the presence of carbon, nitrogen, and oxygen. In the C 1 s spectrum, four peaks are observed at 284.59, 285.85, 286.73, and 288.93 eV, corresponding to single bonds C-C, C-N, C-O, and O=CO, respectively [25,26,27]. The O 1 s spectrum shows three peaks, with two being attributed to double-bond C=O (532.18 eV) and single-bond C-O (530.60 eV) [28,29]. Upon activation, the activated carbon underwent a thorough water rinsing process to eliminate any soluble components. Despite the alkaline nature of the activators used, they were completely washed away due to their high solubility in water. Previous research [30] has indicated that during the activation process, carboxyl and hydroxyl groups are formed on the carbon surface, acting as Brønsted acids with a low pKa, thus displaying acidic properties. Additionally, the carbonyl groups on the activated carbon are inherently acidic and remain unaffected by the type of activators used. In some instances, the activated carbon may exhibit alkaline characteristics, but this can be attributed to the presence of residual activators.

#### 2.1.4. XRD

**Figure 4:**
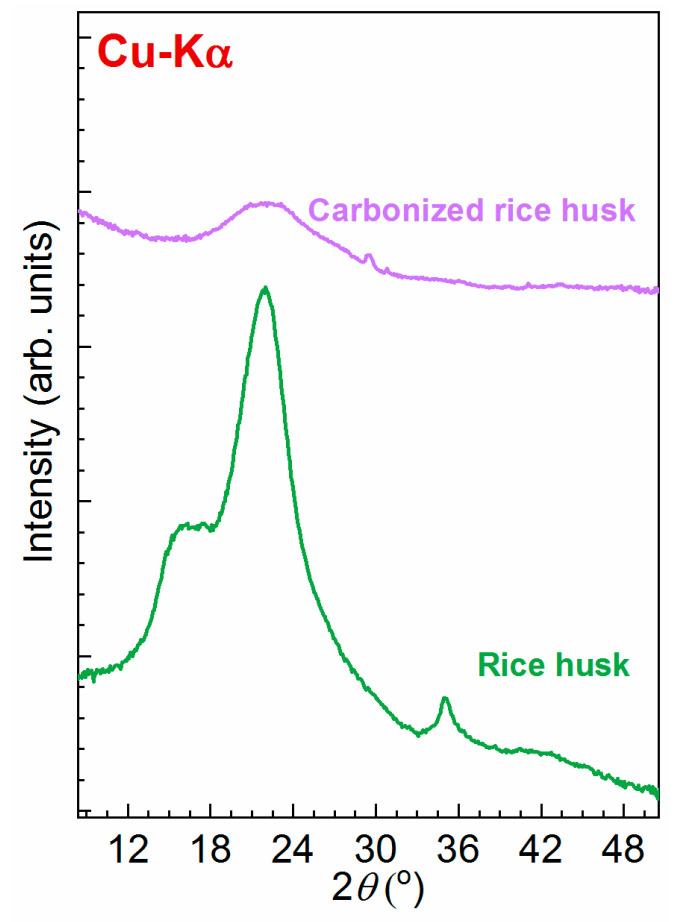
XRD patterns for rice husk and carbonized rice husk.

X-ray diffraction patterns of rice husk, carbonized and activated rice husk are represented in Figure 4. According to previous studies [31], the characteristic diffraction peaks of rice husks are at 2*θ* values of ~16, 22 and 35.1°, which can be attributed to the peaks of cellulose. Additionally, a broad peak in the range of 18–30° is attributed to silica with an amorphous nature. The broad peak observed in the carbonized rice husk between 17 and ~30° is assigned to amorphous carbon [32,33]. In contrast to the previous results, our data clearly do not reveal a wide peak, attributed to the 2*θ* region 42–47° due to high broadening and low intensity. Low-intensity additional peaks on the carbonized rice husk diffraction spectrum refer to the ZnO impurity arising from the synthesis process.

### 2.2. Analysis of Electrochemistry

Activated carbon may be considered as a cathode material for hybrid electrochemical systems such as lithium-ion [34] or lithium-metal capacitors [35]. Therefore, in this work, the electrochemical characteristics of the synthesized material were studied using lithium-conducting electrolytes.

The baseline electrolyte was 1 M solution of LiPF_6_ in mixture of EC:DEC:DMC (1:1:1 vol.), denoted as E1, which is used in traditional lithium-ion batteries and can be considered as an electrolyte for lithium-ion capacitors with a prelithiated graphite anode. The measurements were carried out in symmetrical 2032 coin cells with electrodes based on synthesized activated carbon.

When considering the possibility of using activated carbon in lithium-metal capacitors, it is necessary to take into account that the electrolyte must both ensure the electrochemical stability of the carbon cathode material in the operating voltage range and contribute to the formation of a homogeneous mechanically stable SEI (solid electrolyte interphase) with high ionic conductivity, resulting in uniform lithium plating during charge.

It is generally accepted that the presence of fluorine-containing compounds in the SEI structure affects its mechanical strength, while the Li_3_N-rich protection layer contributes to an increase in ionic conductivity, thus resulting in dendrite growth suppressions [36,37,38]. Therefore, electrolytes containing such additives as FEC (fluoroethylene carbonate) or LiNO_3_ and LiDFOB (lithium difluoro(oxalato)borate) as salts provide prolonged cycling of the lithium metal electrode at a relatively high Coulombic efficiency [39,40,41].

In this work, activated carbon was tested in 2032 cells with lithium metal anode and 1 M solution of LiDFOB in mixture FEC:DME (3:7 vol.) + LiNO_3_ 3%wt. as the electrolyte (denoted as E2).

Note that the study of the influence of the electrolyte composition on the morphology of the lithium electrode during cycling is beyond the scope of this work. Therefore, the main emphasis was placed on the demonstration of possible applicability of activated carbon in hybrid power sources.

**Figure 5:**
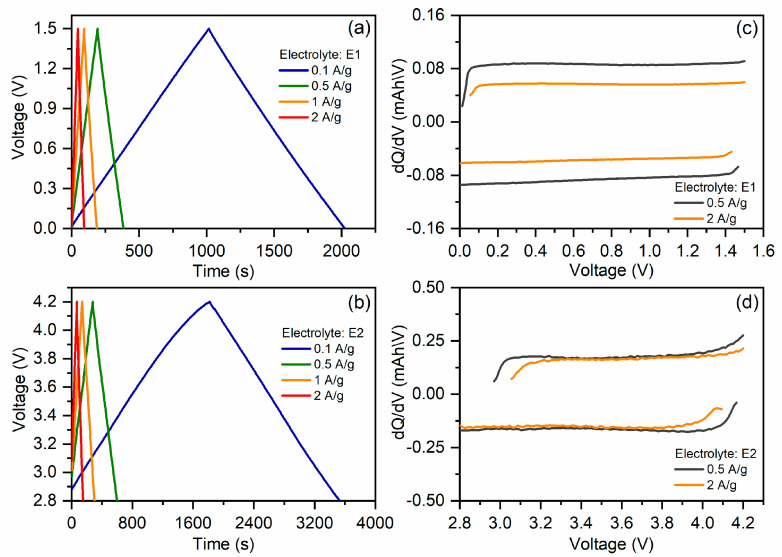
Galvanostatic charge/discharge curves of symmetric coin cell with electrolyte E1 (**a**) and coin cell with metallic lithium anode and electrolyte E2 (**b**). Differential capacity of symmetric coin cell (**c**) and coin cell with metallic lithium anode (**d**).

The profiles of the galvanostatic and differential capacity curves of coin cells presented in Figure 5 indicate the absence of significant redox processes on the surface of the carbon electrodes in both electrolytes in the given voltage ranges. The electrode capacitance occurs mainly due to the formation of an electrical double layer.

**Figure 6:**
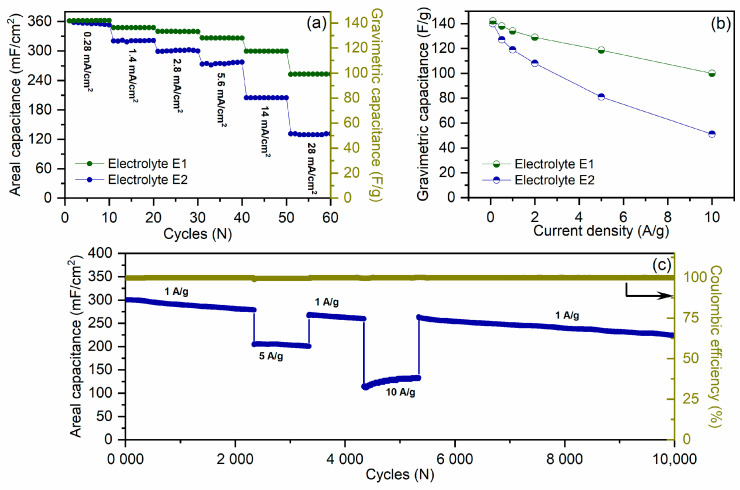
C-rate performance of activated carbon-based electrodes in different electrolytes (**a**). Gravimetric capacitance of the electrodes in different electrolytes at different current densities (**b**). Cycling stability of the coin cell with lithium anode and electrolyte E2 (**c**). Areal capacitance was calculated taking into account the area of the electrode. Gravimetric capacitance was calculated taking into account mass of activated carbon in single electrode.

At a discharge current of 0.1 A/g (0,28 mA/cm^2^), the areal capacitance of the electrodes in both electrolytes is approximately of 360 mF/cm^2^, which, taking into account the mass fraction of activated carbon in the electrode layer, corresponds to a gravimetric capacitance of ~140 F/g (Figure 6a). Increasing the discharge current up to 10 A/g (28 mA/cm^2^) leads to the electrode capacitance retention to 100 F/g (253 mF/cm^2^) in the electrolyte E1 and to 51 F/g (129 mF/cm^2^) in the electrolyte E2 (Figure 6a,b).

Despite the fact that the ionic conductivity of both electrolytes is nearly the same (see Supplementary Materials), such a significant difference in electrode C-rate performance may be associated with the smaller size of the PF_6_^−^ anion in the electrolyte E1 compared with the DFOB^−^ anion in the electrolyte E2 [42,43], as well as with the presence of charge transfer resistance on the SEI-covered lithium electrode.

Figure 6c shows the cycling stability of the coin cell with a lithium anode, activated carbon-based cathode, and electrolyte E2. It can be seen that the areal capacitance decay is approximately 25% after 10,000 cycles. SEM postmortem analysis of the cathode before and after cycling did not reveal any significant structural changes in the electrode surface (not presented here).

A comparative analysis of the differential capacity of the coin cell after the 100th and 10,000th cycles showed that even after a prolonged cycling, the electrode capacitive properties were mainly associated with the formation of an electrical double layer and no significant side redox processes could be observed (see Supplementary Materials). The results indicate the electrochemical stability of the cathode material during cycling in the voltage range of 2.8–4.2 V, and the degradation observed during cycling can be attributed primarily to the side reactions on the lithium surface.

Our comparative analysis with the electrochemical characteristics of other activated carbons obtained from various raw biomaterials for lithium-ion capacitors also showed that the proposed in this work material has excellent capacitive and cycling properties (Table S1 in Supplementary Materials).

Thus, the presented data make it possible to consider the synthesized activated carbon as a promising electrode material in hybrid power sources. However, the prototyping of full-scale devices is associated with further optimization of an electrolyte content and electrode microstructure.

## 3. Materials and Experimental Methods

### 3.1. Synthesis of Activated Carbon from Rice Husk

Rice husk (RH) was collected from a field in the Almaty region (Kazakhstan), and utilized as the low-cost carbon precursor for the synthesis of activated carbon. RH was washed several times with boiled tap water and then with pure filtered water in order to remove dirt and dust particles. Wet RH was dried in a laboratory drying chamber at a temperature of 110 °C for 10–12 h. Drying time depends on several factors such as the amount of wet RH and the type of drying chamber.

The carbonization of dried RH was conducted using cylindrical carbon rich steel reactor at the temperature of 750 ± 2 °C for 90–100 min under nitrogen atmosphere. Nitrogen gas flow rate was approximately 250–350 cm^3^/min (sccm). The temperature in the furnace was automatically raised to 750 °C at a heating rate of 7.5 °C/min with a PID programmable temperature controller. Once the desired temperature was reached, the temperature was maintained at 750 ± 2 °C for 90–100 min. No special attention was paid to the control of the cooling rate. In this study, the carbonization temperature is higher than the usual carbonization temperature of 450–550 °C [44,45]. Biomass carbonization starts at 450 °C; however, we intentionally raised the temperature to 750 °C. At a temperature of 750 °C, carbonized rice husk begins the process of graphitization. The main reason for graphitization occurring in rice husk is the presence of silica. On average, rice husk typically contains around 15–20% silica (SiO_2_) by weight. The silica content in rice husk can vary depending on various factors such as the rice variety, growing conditions, and processing methods [46]. SiO_2_ in RH can act as a catalyst or template for graphitization of amorphous or non-graphitic carbon due to its ability to interact with carbon atoms and provide a suitable environment for graphitic growth [47,48]. The mechanism involves the rearrangement of carbon atoms into a more ordered, layered structure to form graphitic carbon. The exact mechanism of graphitization with SiO_2_ is not fully understood.

The final step, thermochemical activation, was performed by mixing carbonized–graphitized RH with potassium hydroxide powder in an optimized proportion of 1 part carbonized–graphitized RH to 4 parts potassium hydroxide. To ensure complete impregnation, the resultant mixture was subjected to heat inside a heating chamber, at a temperature of 150 °C. The mixture was maintained at this temperature for two hours. Following that, the prepared mixture underwent a thermal activation process under an inert gas atmosphere at a specific heating temperature of 850 ± 5 °C. Argon gas (first grade 99.987%) was used as an inert gas, which had a flow rate of approximately 250 sccm. The pressure in the reactor was maintained at 1 atm. This temperature was maintained for a period of 1.5 h. The temperature of the cylindrical stainless steel reactor was gradually increased at a rate of 9.5 °C per minute until it reached the final desired temperature. In order to prevent oxidation of carbonaceous substance, a protective inert atmosphere was maintained within the reactor. This was achieved by continuously supplying Ar gas at a flow rate of 250 sccm. Once the thermochemical activation process was completed, the obtained samples were rinsed with hot distilled water. This washing process aimed to remove potassium compounds from the samples until reaching a pH value of approximately 6–7. Subsequently, the samples were subjected to drying. Initially, they were dried in ambient air at a temperature of 120 °C for 10 h, followed by vacuum drying step at a temperature of 150 °C, which lasted for 2 h [49,50].

### 3.2. Characterization Methods

The study of the obtained carbon structures on a catalytic nickel substrate was carried out on an Raman spectrometer (NTEGRA Spectra, NT-MDT, Shanghai, China) at a wavelength λ = 473 nm. The surface of the obtained graphene structures on a nickel substrate was examined using a JEOL scanning electron microscope (SEM) (model JSM-6490LA, JEOL Ltd., Tokyo, Japan, accelerating voltage from 0.1 to 30 kV, probe diameter up to 3.0 nm, magnification from ×5 to ×300,000) according to the standard methodology. X-ray photoelectron spectroscopy (XPS) measurements were conducted on a instrument (model: Mutilab ESCA 3000, VG-Microtech, London, UK) equipped with a 9 channeltrons hemispherical electron analyzer and X-ray radiation source with Mg and Al anodes. The XPS spectra were calibrated using the C 1 s peak at 284.6 eV. Shirley-type background subtraction was applied to remove the background signal. The spectra were fitted using a nonlinear least-squares method employing a combination of Gaussian and Lorentzian line shapes. CasaXPS software 2.3.25 was used for the data analysis. The specific surface area of the obtained samples was studied on a SorbtometrM analyzer (ZAO Katakon, Novosibirsk, Russia) and an analyzer ASAP 2400 V3.07” (Micromeritics Instrument Corp., Norcross, GA, USA). XRD measurements were carried out using the Xeuss 3.0 SAXS/WAXS System (Xenocs SAS, Grenoble, France) operating in point geometry equipped with a GeniX^3D^ microfocus generator of X-ray radiation with source Mo-Kα (λ = 0.71078 Å). The spectrometer was equipped with a moving detector Eiger2 R 1 M 2D-detector (Dectris, Baden, Switzerland) with a sensitive area of 77.1 × 79.7 mm^2^ and integrated with the XSACT program. The measurements performed using a 46 mm sample-to-detector distance make it possible to obtain the X-ray scattering intensity I(q) in the range of momentum transfer of approximately ~1< q < ~8 Å^−1^. The measurements were carried out in vacuum at room temperature in the borosilicate glass capillaries (Hilgenberg, Germany, 0.1 mm wall thickness, 1.5 mm flight path).

### 3.3. Electrochemical Tests

For the fabrication of the electrode layers, an electrode slurry was used, which included a mixture of carbon nanotubes, OCSiAl and carbon black, Super C45 as a conductive additive (5% wt.), polyvinylidene difluoride, PVDF (5% wt.) and an active material (90% wt.).

In order to prepare the electrode slurry, PVDF was dissolved in N-methyl pyrrolidone for 2 h at 60 °C under stirring. After complete dissolution of the polymer binder, a pre-mixed sample of the active material and the conductive additive was added and stirred on an overhead mixer for 20 h to form a homogeneous slurry.

Next, the obtained electrode slurry was stirred on a vacuum mixer for 20 min to remove gaseous substances. After that, the slurry was covered on aluminum foil using Doctor Blade technique and dried at 100 °C for 12 h until the solvent completely evaporated. Dry electrode layer areal mass loading was 2.8 mg/cm^2^.

After complete drying, the electrode tape was calendered at a temperature of 100 °C with a compression ratio of 20%. Furthermore, electrodes were laser cut on discs with an area of 1.77 cm^2^ for 2032 coin cells.

The electrodes were weighed and vacuum dried at a temperature of 120 °C for five hours to remove trace amounts of water and transferred to a glove box, where the coin cells were assembled in an argon atmosphere. Polypropylene membrane Celgard 2500 was used as a separator. In some experiments, metallic lithium discs with thickness of 150 µm were used as negative electrodes. Electrochemical measurements were conducted using Neware battery tester at charge/discharge current density varied in a range of 0.1–10 A/g. For the symmetric cell, the voltage range was 0.01–1.5 V. For the cell with lithium anode, the voltage range was 2.8–4.2 V.

The capacitance C was calculated based on galvanostatic charge/discharge curves of coin cells according to the equation:

C = (I × ∆t)/∆V(1)

where I—charge/discharge current; Δt—charge/discharge time; ΔV—voltage window.

Gravimetric (F/g) and areal capacitance (mF/cm^2^) were calculated by dividing the capacitance C by the mass fraction of the active material in the electrode layer and the electrode area, respectively.

The measurements of electrolyte ionic conductivity were carried out in electrochemical cells with polished stainless steel blocking electrodes using the electrochemical impedance spectroscopy (EIS) method. The frequency range was 0.5 Hz–1 MHz. Voltage amplitude was 10 mV. Area of the blocking electrode was 0.79 cm^2^. Distance between electrodes was 70 µm. The electrolyte ionic conductivity σ was calculated in accordance with the following equation:

σ = d/(R × S)

were d—distance between blocking electrodes; S—area of the blocking electrode; and R—the bulk resistance which is the value corresponding to the intersection point of Nyquist curves with the real axis from EIS data.

## 4. Conclusions

In summary, the production of activated carbon through chemical activation of lignocellulosic biomass, specifically rice husks, offers a promising pathway for sustainable and efficient resource utilization. The incorporation of potassium hydroxide as the activation agent results in activated carbon with remarkable structural and adsorptive properties. XRD measurements confirmed the amorphous nature of the sample. XRD has been found to be a useful technique for activated carbon (AC) studies.

The results of electrochemical measurements showed that the specific capacitance of the synthesized material in lithium-conducting electrolytes is about 140 F/g. At the same time, prolonged cycling of coin cells with metallic lithium anode showed that activated carbon-based electrodes have a relatively high stability in the voltage range of 2.8–4.2 V. This allows us to consider synthesized activated carbon as a promising electrode material for lithium-ion and lithium-metal capacitors.

For lithium-metal capacitors, our estimations reveal that utilization of metallic lithium as anode and rice-husk activated carbon as a cathode material may significantly reduce mass of the capacitor and increase cell voltage which in general results in energy density increase up to 35 Wh/kg.

## Data Availability

All the data relevant to the work are included in the manuscript.

## Supplementary Materials

The following supporting information can be downloaded at: https://www.mdpi.com/article/10.3390/molecules28155818/s1, Figure S1. SEM image of RH-derived activated carbon; Figure S2: Ionic conductivity of the electrolytes; Figure S3: Differential capacitance after 100th and 10,000th cycles; Table S1. Comparison of specific capacitance and electrochemical test conditions for different activated carbons.
