# Supplementary material for: Characterization of Activated Carbon from Rice Husk for Enhanced Energy Storage Devices
Source: Molecules. 2023 Aug 2;28(15):5818. doi: 10.3390/molecules28155818 (PMC10421275; doi:10.3390/molecules28155818)
Supplement: Supplementary file 1 [file molecules-28-05818-s001.zip › molecules-2508019-supplementary.pdf]

SUPPLEMENTARY MATERIALS

Characterization of Activated Carbon from Rice Husk for Enhanced Energy Storage Devices

Meir S. Yerdauletov [1,2,3], Kuanysh Nazarov [1,2,*], Bagdaulet Mukhametuly [1,2,4], Mukhtar A. Yeleuov [1,5], Chingis Daulbayev [1,6], Roza Abdulkarimova [4], Almas Yskakov [1,2,3], Filipp Napolskiy [2,7] and Victor Krivchenko [7]

[1] Institute of Nuclear Physics, Almaty 050032, Kazakhstan
[2] Joint Institute for Nuclear Research, 141980 Dubna, Russia
[3] Faculty of Physics and Technics, L.N. Gumilev Eurasian National University, Astana 010008, Kazakhstan
[4] Faculty of Physics and Technology, Al-Farabi Kazakh National University, Almaty 050040, Kazakhstan
[5] Bes Saiman Group, Almaty 050057, Kazakhstan
[6] National Laboratory Astana, Nazarbayev University, Nur-Sultan 010000, Kazakhstan
[7] Battery Prototyping Laboratory, Dubna State University, 141982 Dubna, Russia
* Correspondence: knazarov@jinr.ru

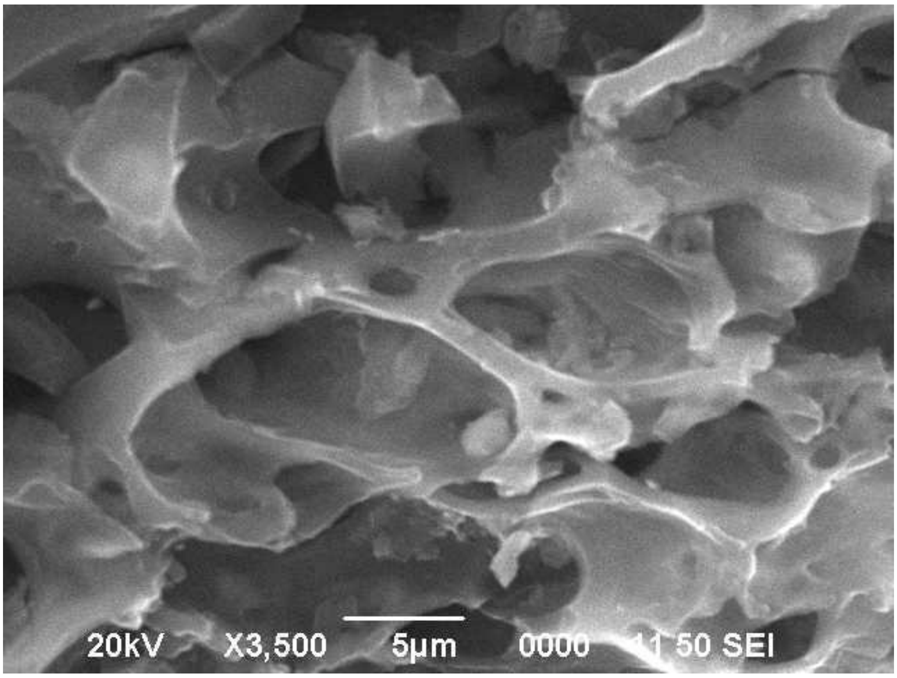

Figure S1. SEM image of RH-derived activated carbon.

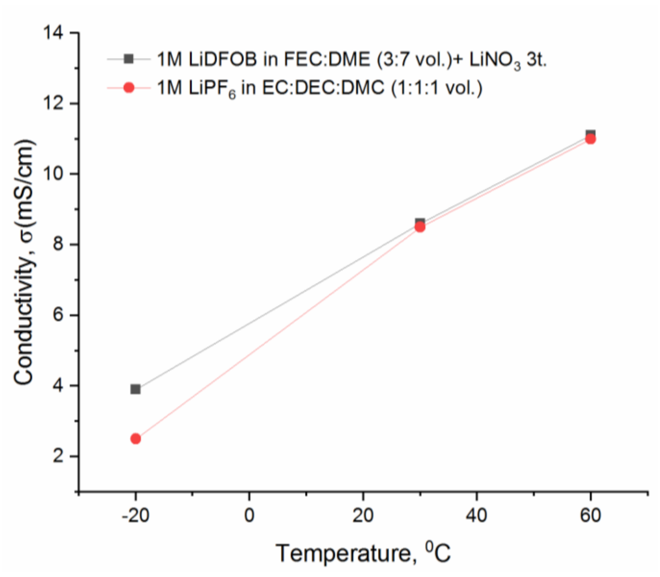

Figure S2. Ionic conductivity of the electrolytes.

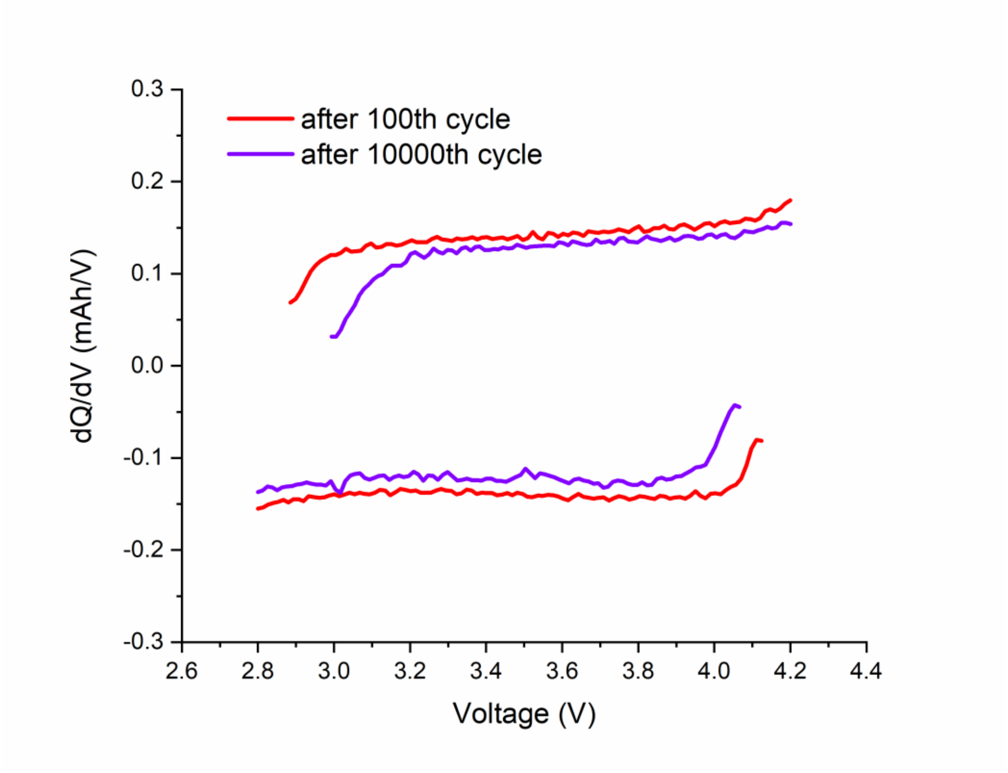

Figure S3. Differential capacity after 100th and 10000th cycles.

Table S1. Comparison of specific capacitance and electrochemical test conditions for different activated carbons.

Raw material	Negative electrode in electrochemical cell	Electrolyte	Specific capacitance, F/g	Cycles, N	Capacitance (Energy density) decay after cycling, %	Source
Rice husk	Li	1M LiDFOB in FEC:DME (3:7 vol.)+LiNO3 3%wt.	140	10000	25	This work
Coconut shell	$Li_4Ti_5O_{12}$	1M $LiPF_6$ in EC:DMC (1:1)	159	2000	near 15	DOI: 10.1038/srep03002
Teakwood sawdust	$Li_4Ti_5O_{12}$ or LiC_6	1M $LiPF_6$ in EC:DMC (1:1)	131	2000	near 15-30	https://doi.org/10.1016/j.electacta.2017.01.060
Human hair	$Li_4Ti_5O_{12}$	1M $LiPF_6$ in EC:DMC (1:1)	115	1000	15-20	http://dx.doi.org/10.1016/j.electacta.2015.09.127
Polymer derived high surface area porous carbon	$Li_4Ti_5O_{12}$	1M $LiPF_6$ in EC:DMC (1:1)	123	2000	near 20	http://dx.doi.org/10.1016/j.electacta.2014.03.079
Orange Peel	$Li_4Ti_5O_{12}$ or LiC_6	1M $LiPF_6$ in EC:DMC (1:1)	112.5	2500	15-40	DOI: 10.1002/slct.201700574
Rice Husk	$Li_4Ti_5O_{12}$	1M $LiPF_6$ in EC:DMC (1:1)	120	2000	10	http://dx.doi.org/doi:10.1016/j.electacta.2016.06.055
